# Clinical Outcomes of FP-7/8 Ahmed Glaucoma Valves in the Management of Refractory Glaucoma in the Mainland Chinese Population

**DOI:** 10.1371/journal.pone.0127658

**Published:** 2015-05-21

**Authors:** Yingting Zhu, Yantao Wei, Xuejiao Yang, Shuifeng Deng, Zuohong Li, Fei Li, Yehong Zhuo

**Affiliations:** State Key Laboratory of Ophthalmology, Zhongshan Ophthalmic Center, Sun Yat-sen University, Guangzhou, People’s Republic of China; Bascom Palmer Eye Institute, University of Miami School of Medicine;, UNITED STATES

## Abstract

**Background:**

To evaluate the efficacy and safety of the Ahmed glaucoma valve (AGV) and the risk factors associated with AGV implantation failure in a population of Chinese patients with refractory glaucoma.

**Method:**

In total, 79 eyes with refractory glaucoma from 79 patients treated in our institution from November 2007 to November 2010 were enrolled in this retrospective study. The demographic data, preoperative and postoperative intraocular pressures (IOPs), best corrected visual acuity (BCVA), number of anti-glaucoma medications used, completed and qualified surgery success rates and postoperative complications were recorded to evaluate the outcomes of AGV implantation. Factors that were associated with implant failure were determined using Cox proportional hazard regression model analysis and multiple linear regression analysis.

**Principle Findings:**

The average follow-up time was 12.7±5.8 months (mean±SD). We observed a significant reduction in the mean IOP from 39.9±12.6 mm Hg before surgery to 19.3±9.6 mm Hg at the final follow-up. The complete success rate was 59.5%, and the qualified success rate was 83.5%. The number of previous surgeries was negatively correlated with qualified success rate (P<0.05, OR=0.736, 95% CI 0.547-0.99). Patients with previous trabeculectomy were more likely to use multiple anti-glaucoma drugs to control IOP (P<0.01). The primary complication was determined to be a flat anterior chamber (AC).

**Conclusion:**

AGV implantation was safe and effective for the management of refractory glaucoma. Patients with a greater number of previous surgeries were more likely to experience surgical failure, and patients with previous trabeculectomy were more likely to use multiple anti-glaucoma drugs to control postoperative IOP.

## Introduction

In recent decades, glaucoma has become the leading cause of irreversible blindness [1–3]. In China, among 9.4 million people at least 40 years of age suffering from glaucomatous optic neuropathy, 5.2 million (55%) are blind in at least one eye and 1.7 million (18.1%) are blind in both eyes [4]. It is predicted that the number of people with bilateral blindness will increase to 5.9 million and 5.3 million for people with open-angle glaucoma (OAG) and angle-closure glaucoma (ACG), respectively, by 2020 [5].

Patients with refractory glaucoma are unresponsive to routine treatment, including medical treatment for lowering intraocular pressure (IOP) and traditional surgical procedures. Most cases of refractory glaucoma consist of secondary glaucoma with complex features, including very high IOP, various and/or unknown mechanisms for elevated IOP, limited assessment and treatment due to perplexing ocular factors, the need for combination therapies, poor prognosis and rapid deterioration in vision [5].

Considering the higher risk of failure with conventional filtering surgery, the implantation of glaucoma drainage devices is frequently performed in patients with glaucoma that is intractable to drug therapy or trabeculectomy. Moreover, it has become the priority treatment for a variety of situations, such as neovascular glaucoma (NVG), iridocorneal endothelial syndrome (ICE), penetrating keratoplasty with glaucoma, glaucoma following retinal detachment surgery [1], and glaucoma associated with aphakia or pseudophakia, trauma, uveitis and vitreoretinal disorders [6].

The AGV is one of the available glaucoma drainage devices (GDDs) for aqueous humor drainage, and it was introduced in 1993 [7]. The silicone AGV (FP-7/8) is a built-in Venturi valve that is formed by a folded silicone elastomer membrane with a free edge permitting only one-way outflow due to set resistance to the aqueous humor [8]. This type of GDD was approved by the Chinese Food and Drug Administration (CFDA) in November 2007. In its clinical pre-testing period, we compared the implantation of FP-7 and S-2 Ahmed glaucoma valves in refractory glaucoma patients over a short-term follow-up [9]. The AGV was introduced in mainland China much later than in other developed countries; thus, there is little comprehensive information about the efficacy and safety of the AGV (FP-7/8) or concerning long-term follow-up data from Mainland Chinese patients. In this retrospective study, we evaluated the surgical outcomes of AGV (FP-7/8) implantation in patients with refractory glaucoma using the medical records from our institution between November 2007 and November 2010.

## Methods

The present retrospective study was approved by the Zhongshan Ophthalmic Center Institutional Review Board of Sun Yat-sen University because the data were recorded anonymously and analyzed retrospectively. The recruited patients were enrolled from the same academic department (Glaucoma) in our center. Since November 2007, we have used the new FP-7/8 AGV model. The enrollment period was 3 years after the hospital formulary conversion date in November 2007.

### Enrollment and exclusion criteria

We reviewed the medical records from November 2007 to November 2010 for patients with refractory glaucoma that was uncontrolled by medication and required AGV implantation (FP-7/8). Seventy-nine patients (79 eyes) were included from this single department. The exclusion criteria included the inability to participate in follow-up for an extended period after surgery.

### Data collection

Preoperative data for the enrolled patients were collected, including age, sex, detailed clinical history, diagnosis, IOP measured by Goldmann applanation tonometer (GAT) at the first hospital visit, best corrected visual acuity (BCVA) measured with a Snellen chart, slit-lamp examination (SLE) data, the number of anti-glaucoma medications used, the number and type of previous ocular surgeries, and history of other ocular or systemic diseases.

Postoperative data were collected during follow-up, including IOP, BCVA, and SLE data, the number of anti-glaucoma medications used at the previous follow-up visit, follow-up duration and surgical complications. The patients were examined postoperatively at 1 day, 1 week (5–10 days), 1 month (25–35 days), 3 months (70–110 days), and 6 months (160–200 days) and then every 6 months (250–290 days, 340–380 days) thereafter.

### Surgical procedures and evaluation criteria

The surgical procedures and surgical success criteria were outlined in our previously published paper [9], The primary outcome was IOP. Complete surgical success was defined as follows: (1) IOP≥6 mmHg and ≤21 mmHg; (2) IOP reduction of at least 30% relative to preoperative values; and (3) no additional surgical intervention for IOP control, loss of light perception, or serious complications. Qualified success was defined as follows: necessity for the patients to use supplemental anti-glaucoma medication to control the uncontrolled IOP. Failure was defined as follows: eyes requiring further glaucoma surgeries (including cyclophotocoagulation), removal of the implant, and complete loss of light perception during the follow-up. AGV implantation was the established standard of care treatment in our institution for all patients requiring and suited for this surgery. The clinical outcomes included BCVA, mean number of anti-glaucoma medications and complications with suitable management after surgery during the follow-up period. The definitions for hypotony and serious complications were reported previously [9]. Both topical and systemic anti-glaucoma medicines were assessed. The number of medications was calculated by the addition of each medication as one unit, with fixed combinations calculated as two medications.

### Statistical analysis

For comparison between the preoperative and postoperative data, Student’s paired t test with the Bonferroni correction was used for parametric data (IOP), Wilcoxon’s paired signed rank test was used for nonparametric data (number of medications, BCVA), and Fisher’s exact test was used for distributions (sex and complications). A Cox proportional hazards regression analysis was performed to identify potential preoperative characteristics associated with complete or qualified surgical success rate. The glaucoma cumulative survival rate was calculated using a Kaplan-Meier survival estimate. Multiple linear regression analysis was performed to analyze the factors related to the use of a greater number of postoperative anti-glaucoma drugs. Statistical significance was defined as P≤0.05. The statistical analysis was performed with SPSS software, version 13.0 (SPSS, Inc., Chicago, IL, USA).

## Results

### Preoperative characteristics

The mean follow-up period was 12.7±5.8 months (range of 6–26 months). The demographic and preoperative data for the 79 subjects are summarized in Table 1. The average age of the patients was 44.9±15.6 years old (range, 11–73). Twenty-three patients were male, and 56 were female. Fifty-five patients had a history of at least one previous surgery, which was most commonly trabeculectomy (43 patients), while the others underwent phacoemulsification, keratoplasty, or retinal detachment surgery. Approximately 87.4% of the total group of patients had no more than 2 surgeries before AGV implantation, whereas 27.9% had one prior surgery, and 29.1% had two prior surgeries. Among the enrolled eyes, 67 had accepted autogeneic sclera as the cover beneath the exposed sclera tract entrance. The preoperative BCVA results showed that nearly half of all of the patients were classified as having poor vision (<20/400), and 3 patients had already suffered from loss of light perception.

**Table 1:** Demographic and preoperative data of patients treated with the FP-7/8 silicone AGVs.

Variables	FP-7/8 AGV (n = 79)
Eye (OD/OS)	41/38
Age (years, mean ± SD)	44.9±15.6
Range (years)	11–73 (45.0)
Sex (n, male/female)	23/56
Previous trabeculectomy (n)	43
Total previous surgeries (n)	55
Previous surgeries (n)	
≥4times	3
3 times	7
Twice	23
Once	22
None	24
Autogeneic/allogeneic sclera	67/12
Preoperative visual acuity(	
20/20 to 20/40	18
20/50 to 20/80	9
20/100 to 20/400	11
<20/400	36
NLP	3
Follow-up time (months, mean ± SD)	12.7±5.8
Range (months)	6–26

AGV = Ahmed glaucoma valve, SD = standard deviation, NLP = none light perception

The most common preoperative diagnoses were NVG, OAG (including primary open-angle glaucoma, steroid-induced glaucoma and juvenile glaucoma), and primary PACG (post-phacoemulsification), which occurred in 28%, 23% and 13% of the enrolled patients, respectively. The remaining diagnoses included traumatic glaucoma (n = 14), ICE syndrome (n = 7), post-keratoplasty/retinal detachment surgery (n = 4), Axenfeld-Rieger syndrome (n = 1) and congenital glaucoma (n = 2).

### Intraocular pressure (IOP)

**Fig 1:**
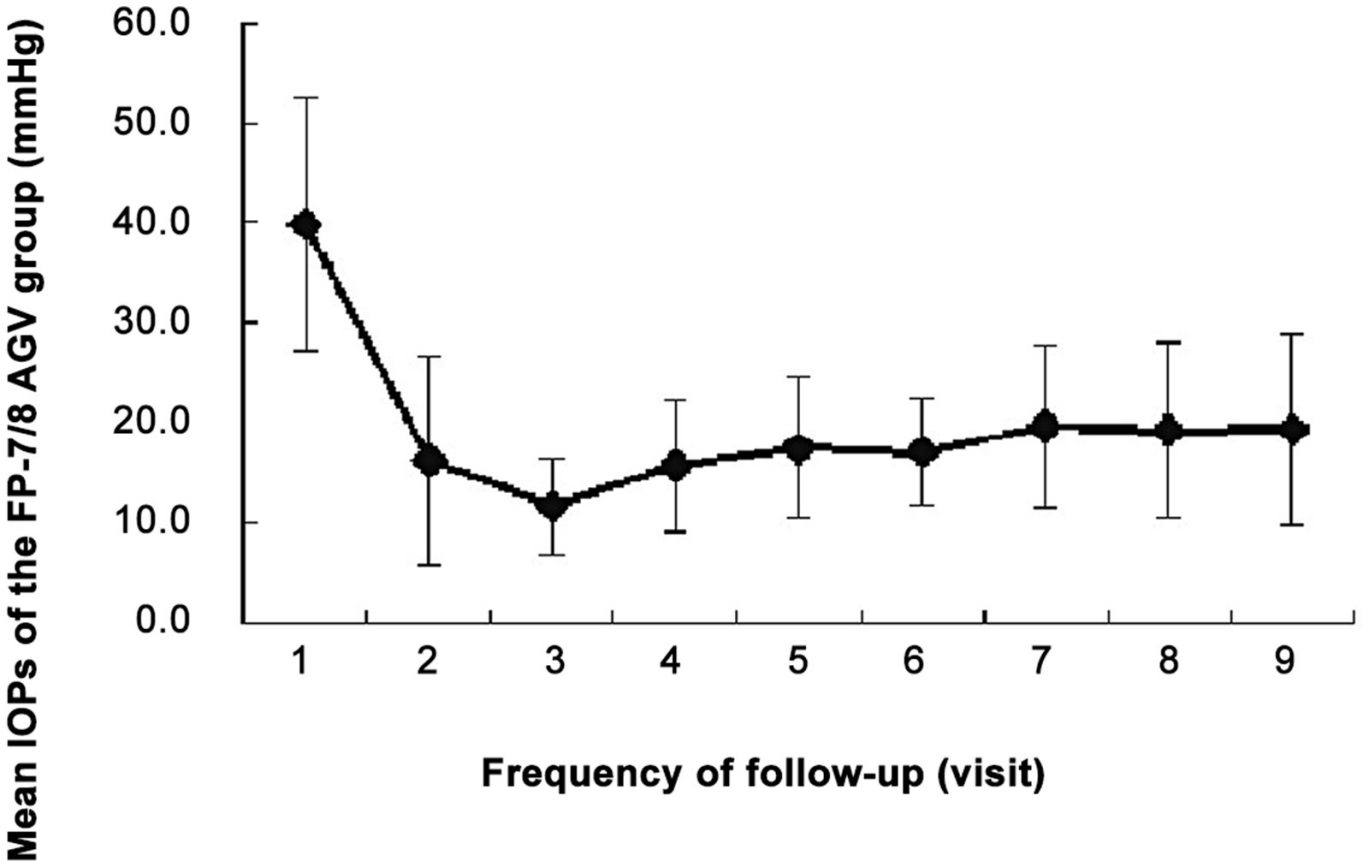
Intraocular pressure (IOP) at baseline and follow-up. Data are presented as the mean± standard error of the mean.

Fig 1 and Tables 2 and 3 show the mean IOP during the follow-up period. The mean preoperative IOP was 39.9±12.6 mm Hg (ranged from 21.5 to 71 mm Hg). On the first day after surgery, the IOP significantly decreased to 16.2±10.5 mm Hg (P<0.05). The IOP was 11.7±4.8 and 15.7±6.6 mm Hg at 1 week and 1 month after surgery, respectively. The lowest average IOP occurred approximately one week after surgery. A small increase in IOP occurred over the first postoperative month, subsequently plateauing until the last follow-up visit (mean 19.3±9.6 mm Hg, range from 8.0 to 58.4 mmHg). There was a significant decrease in IOP at the most recent follow-up visit compared with the preoperative level (P<0.05).

**Table 2:** Comparison of IOP in FP-7/8 AGV patients preoperatively, within one week and during follow-up (mm Hg, mean ± SD).

		Follow-up (days)							
Groups	Preoperative	1	5–10	25–35	70–110	160–200	250–290	340–380	Most recent visit
FP-7/8 AGV (n = 79)	39.9±12.6	16.2±10.5	11.7±4.8	15.7±6.6	17.5±7.1	17.1±5.4	19.6±8.2	19.2±8.2	19.3±9.6
No. of eyes	79	79	77	67	62	58	33	30	79
*P value		<0.001	<0.001	<0.001	<0.001	<0.001	<0.001	<0.001	<0.001

*IOP = intraocular pressure, AGV = Ahmed glaucoma valve, SD = standard deviation

*all compared with preoperative IOP, Student’s paired t test, with Bonferroni correction.

**Table 3:** Preoperative and postoperative comparisons in FP-7 AGV patients.

Variables	FP-7 AGV (n = 79)
IOP (mm Hg, mean ± SD)	
Preoperative	39.7±12.7
Range (median)	21.5–71.0 (40.0)
Postoperative	19.1±9.6
Range (median)	8.0–58.4 (16.0)
Glaucoma medications (range, median)	
Preoperative	0–7 (3)
Postoperative	0–4 (1)
Postoperative BCVA (n)	
Improved or worse within 1 line	53
Worse by more than 1 line	20
Surgical outcome by complete success definition (%)	
Success	59.5
Failure	40.5
Surgical outcome by qualified success definition (%)	
Success	83.5
Failure	16.5

IOP = intraocular pressure, AGV = Ahmed glaucoma valve, SD = standard deviation, BCVA = best corrected vision acuity

### Number of anti-glaucoma medications

As shown in Table 3, the median number of anti-glaucoma medications required before surgery was 3 (range, 0–7), while at the last follow-up visit, the median number was 1 (range, 0–4). There was a statistically significant difference between the number of mediations required preoperatively and at the most recent follow-up visit (P<0.05).

### Visual acuity

At the last follow-up visit, 53 of the enrolled patients showed improved BCVA or no more than a 1-line decline, based on the measurement error of Snellen charts. Twenty patients showed a decrease in vision of more than 1 line. Unfortunately, the record of visual acuity at the last follow-up visit was misplaced in 6 patients. Among all of the patients, 3 exhibited a loss of light perception before surgery, and 4 additional patients reported loss of light perception at the last follow-up visit.

### Surgical success

**Fig 2:**
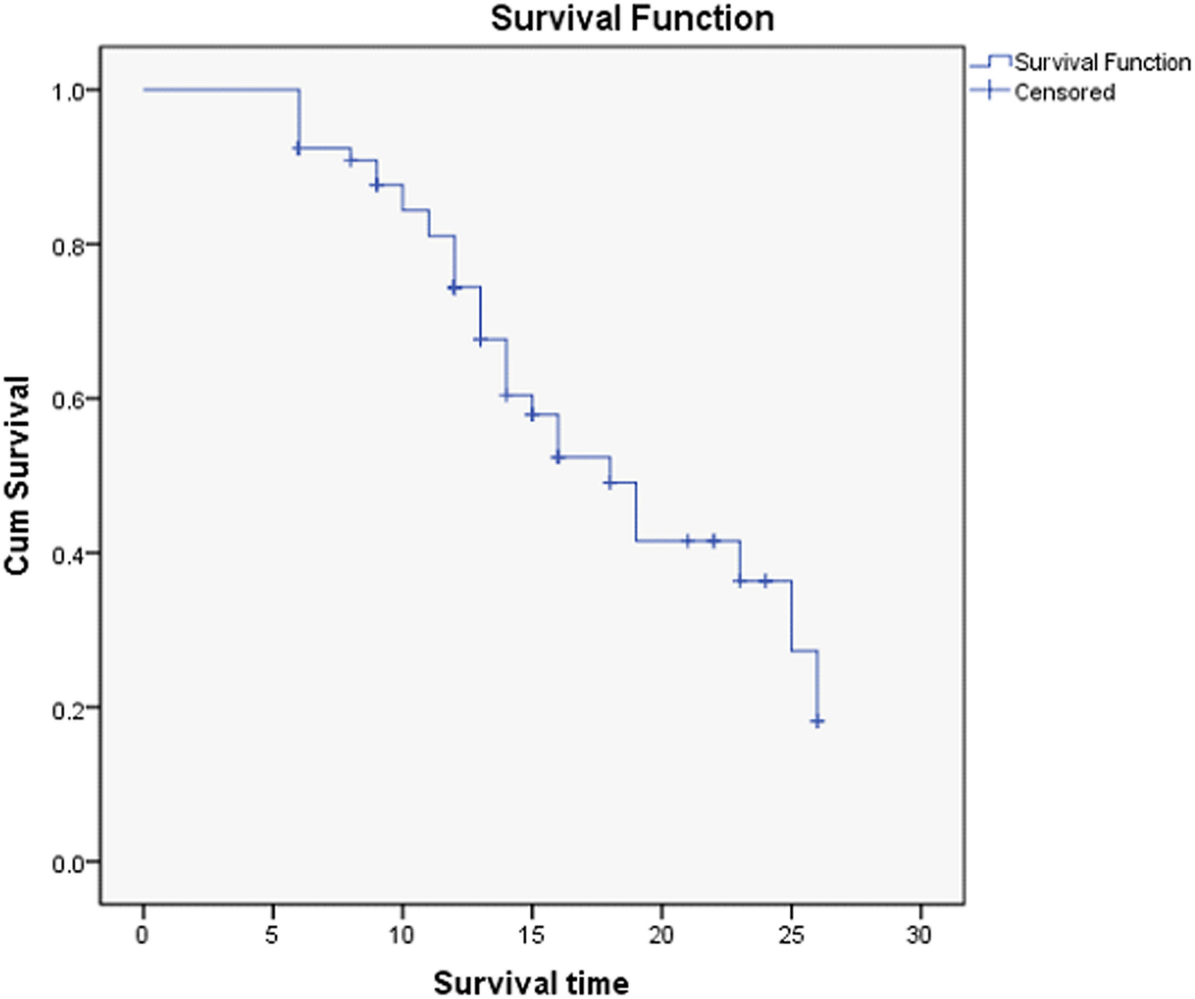
Cumulative survival curves for FP7/8 AGV implant.

The surgical success rates are listed in Tables 3 and 4. The cumulative complete success rate calculated by Kaplan-Meier survival analysis is presented in Fig 2.

**Table 4:** The complete success rate of patients with various glaucoma diagnoses.

Diagnosis	Count (n/total)	Success rate (%)
ICE syndrome	6/7	85.7
OAG (POAG, steroid-induced, juvenile)	10/17	58.8
Neovascular glaucoma	14/24	58.3
Traumatic glaucoma	8/14	57.1
Others (post-keratoplasty/retinal detachment surgery, congenital glaucoma, Alexander-Rieger’s syndrome)	4/7	57.1
PACG (post-phacoemulsification)	5/10	50.0

ICE = iridocorneal endothelial, OAG = open angle glaucoma, POAG = primary open angle glaucoma, PACG = primary angle closure glaucoma

According to the definitions of complete and qualified success, 66 of 79 (59.5%) patients achieved complete success by the last follow-up visit, and the qualified success rate was 83.5%.

As shown in Table 4, the complete success rate was different for each type of glaucoma. The highest statistically significant success rate included 6 patients diagnosed with ICE who achieved complete success after AGV implantation.

### Surgical complications and additional surgeries

Surgical complication details are summarized in Table 5. In total, 23 of 79 patients had surgical complications. The most common type of complication was flat AC. Eight patients developed flat ACs after AGV implantation, and 7 of these patients required reformation. Furthermore, 7 patients suffered from bleb-related complications and underwent bleb dissection or repair. Of the remaining patients with complications, 3 had tube-related obstructions, 3 suffered from hyphema that required AC washout, and 2 had corneal lesions that required a contact lens or even corneal transplant. One patient had a conjunctival laceration that was not related to the AGV implantation. No cases of hypotony were documented.

**Table 5:** Postoperative complication of the FP-7 AGV group after implantation(n(%)).

Variables	FP-7 groups
Tube-related	
Obstruction	3(3.8)
Exposure	0(0)
Migration	0(0)
Erosion	0(0)
Non-tube-related	
Flat AC	8(10.13)
requiring reformation	7(8.86)
self-reformation	1(1.27)
Choroidal effusion requiring drainage	0(0)
Hypotony maculopathy	0(0)
Early (within 3 months)	0(0)
Late (after 3 months)	0(0)
Corneal edema, dellen, decompensation	2(2.53)
Hyphema	3(3.80)
requiring AC wash-out	1(1.27)
self-absorbed	2(2.53)
Retinal detachment	0(0)
Vitreous hemorrhage	0(0)
Endophthalmitis	0(0)
Wound leak	0(0)
Bleb-related	7(8.86)
Patients with complications	16(20.25)

AGV = Ahmed glaucoma valve, AC = anterior chamber

### Risk factors of failure

The results of Cox proportional hazard regression model analysis (Table 6) revealed that age, sex, number of preoperative medications, preoperative IOP and previous trabeculectomy were not related to the complete or qualified success rates for AGV implantation. However, the number of previous surgeries was negatively associated with the qualified success rate (P<0.05, OR = 0.736, 95% CI 0.547–0.99). Multiple linear regression analysis showed that patients who underwent trabeculectomy were more likely to use a greater number of anti-glaucoma medications to control IOP after AGV implantation (P<0.01).

**Table 6:** Potential preoperative characteristics that were related with qualified success rate.

	P*	OR	95.0% CI for OR	
			Lower	Upper
previous intraocular surgery (n)	0.043	0.736	0.547	0.990
previous trabeculectomy (n)	0.601	1.239	0.555	2.764
preoperative IOP (mmHg)	0.931	.999	0.973	1.026
gender (male/female)	0.866	0.949	0.520	1.733
age (year)	0.194	1.014	0.993	1.036
pre-AGV anti-glaucoma medication (n)	0.691	1.037	0.868	1.238
NVG	0.107	0.537	0.253	1.143
OAG (POAG, steroid-induced, juvenile)	0.752	0.884	0.410	1.905
PACG	0.243	1.557	0.740	3.275
Traumatic glaucoma	0.220	1.551	0.769	3.130
ICE syndrome	0.369	0.677	0.289	1.587

*Cox proportional hazard regression analysis

## Discussion

GDDs provide an alternative for refractory treatment. Studies have shown that the patterns and trends for glaucoma surgical procedures have changed since 1996. The use of GDDs has increased over time, and the number of trabeculectomies performed has decreased [10, 11]. Unlike earlier drainage implants (Molteno and Baerveldt), which caused numerous postoperative complications with hypotony due to the lack of valves [12, 13], AGVs can safely achieve a comparable IOP-lowering effect. AGVs (FP7/8) were approved by the Chinese Food and Drug Administration (CFDA) in November 2007. Because of the recent increase in the use of AGVs in Mainland China, this retrospective study provided the first comprehensive information about the efficacy and safety of AGVs (FP-7/8) in the Mainland Chinese population, based on long-term follow-up data.

Two years after AGV implantation, the IOP of patients was significantly decreased compared with preoperative values and the most recent follow-up visit, which occurred at an average of approximately 1 year after surgery (Table 2). The average IOP was minimal at the third follow-up (1 week after surgery) and was slightly increased at the fourth follow-up (1 month after surgery); however, the IOP subsequently plateaued until the last follow-up visit. These data were in accordance with our previous study [9, 14] and other reports [6, 12, 13] that showed that silicone FP-7/8 AGVs could effectively control long-term IOP in refractory glaucoma patients (1 to 3 years). Our study also showed that patients with refractory glaucoma suffered from an elevated IOP at 1 month after AGV implantation; therefore, postoperative follow-up is important, and additional anti-glaucoma medications or other interventions should be used to control IOP and prevent visual loss [15].

In our study, the complete success rate was 59.5% at the last follow-up visit, and the qualified success rate was 83.5%. Due to the differences in the enrolled populations, follow-up periods, and success criteria, it is difficult to compare success rates across studies. According to previous reports, the complete success rate of AGV implantation has ranged from 25.2% to 94% [16, 17, 18]. Our complete success rate was in accordance with that of the most recent randomized controlled trial by Panos G. Christakis and associates, which compared the AGV with the Baerveldt (AVB) [13]. After classifying our enrolled population into different types of refractory glaucoma, we found that patients with ICE syndrome achieved the highest complete success rate. Few studies were found with a limited follow-up time after reviewing the reports regarding patients with glaucoma secondary to the ICE syndrome. Our complete success rate (85.7%) for ICE syndrome is slightly higher than the rate (71% at 1 year, 71% at 3 years, and 53% at 5 years) reported by Erin A. Doe, et al. in 2001[19]. In this report, the glaucoma drainage implants were predominantly Baerveldt and Molteno. There was only one patient using the Ahmed implant. Due to the additional valve design of the Ahmed glaucoma implant, there may be fewer postoperative complications. However, because of the limited samples and follow-up time in our study, more patients with ICE syndrome should be recruited, and their long-term outcomes should be analyzed in the future.

Most postoperative complications occurred within 3 months, which was comparable to the AVB study [13]. Many retrospective and prospective studies have shown that hypotony was the most common complication with AGV implantation. Hypotony results from excess drainage due to failure of priming, a broken valve or excessive aqueous drainage from the peritubular insertion site [20, 21]. As previously reported, there were no cases of hypotony [9]. This result was likely due to the use of viscoelastic, which is absorbed within 3–5 days after surgery [22, 23]. With the advantage of filtration-restricted features, hypotony-related complications were decreased, compared with valveless implants [12, 13].

The earliest postoperative complication in our study was a flat AC that required reforming. These data were in accordance with the Ahmed Baerveldt Comparison study by Budenz DL and associates [12]. Not all early flat ACs required aggressive interventions, as they could gradually self-reform. However, a flat AC can result in severe complications, such as corneal edema, dellen and decompensation or inflammation of the iris, due to contact between the tube and the cornea or iris. Thus, AC gonioplasty was performed when necessary.

The second most common postoperative complications were bleb-related, which occurred approximately 3 months after implantation. A higher rate of bleb encapsulation after AGV implantation has been reported, compared with Baerveldt implantation [24]. Differences in plate materials and size [25] have been suggested as causes for bleb development. Previous studies [26, 27], including our report [9], have suggested that silicone FP-7/8 AGVs have a lower rate of encapsulation than polypropylene S-2 valves. However, a moderate (approximately 10%) encapsulation rate was observed in our study and in other retrospective and prospective reports [13, 28]. Alternative hypotheses regarding the cause of encapsulation have been suggested, including fibrosis induced by inflammatory cytokines from the aqueous humor [29]. The use of intraoperative mitomycin yields less fibrosis but higher incidences of hypotony and scleral graft melt [30, 31]. However, no cases of hypotony or scleral graft melt occurred in our study.

In our study, the three patients with postoperative hyphema were all diagnosed with NVG, likely due to the vulnerability of newly formed vessels. Therefore, we should pay close attention during the insertion of the tube into the AC in those patients with NVG. According to recent studies [32–34], neovessels can be treated by photocoagulation or anti-VEGF treatment. We chose conservative treatment for three patients first, consisting of semi-supine rest and less activity; however, in two of the patients (but not in the third), the blood in the AC was absorbed gradually. Consequently, we had to perform an AC wash-out.

As the number of AGV implants gradually increases, the general surgical techniques required will no longer constitute a barrier for senior surgeons. However, techniques are still in need of optimization. Some methods have been created to increase the success rate. For example, Dr. Zhang [35] has found that changing pre-treatment of the sclera bed and overlying Tenon’s tissue/conjunctiva with MMC to pre-treatment of the valve plate with MMC may decrease the incidence of encapsulated cysts and increase the success rate. Moreover, the management of postoperative complications is critical for patients. Thus, a strict preoperative plan, with follow-up visits and patient compliance, is important for refractory glaucoma management.

Our previous study [9] supported other research [6] showing that previous trabeculectomy is a risk factor for AGV implantation failure, whereas other investigators reported NVG [36] and a decreasing pre-operative IOP (per mm Hg) [37] as being risk factors for surgical failure. Traditional surgical management for refractory glaucoma includes trabeculectomy as the first choice, while GDDs are reserved for patients with intractable glaucoma. Previous trabeculectomy can cause additional fibrosis to the conjunctiva around the surgical area [38] and impair the success of AGV implantation. Although our present study showed that patients with past medical histories of trabeculectomy were not at significant risk for AGV implantation failure, these patients were more likely to use additional drugs to control postoperatively elevated IOP.

As reported in a recent study[39], although trabeculectomy and glaucoma tube shunt surgery could achieve similar IOP reduction and usage of additional anti-glaucoma medication postoperatively, the latter procedure had a higher success rate and fewer additional glaucoma surgeries for patients with uncontrolled glaucoma. In our routine clinical practice, we have already considered the higher risk of failure with conventional filtering surgery and have chosen glaucoma drainage device implantation as priority surgical treatment for a variety of situations. For example, one of our recruited patients with NGV used 4 types of anti-glaucoma medication, achieving an IOP of 11.5 mmHg; however, this patient complained of using too many medications every day and was willing to have surgery. Another patient with ICE syndrome was not using any anti-glaucoma medications and had an IOP of 43.3 mmHg. We considered that an AGV implant might save time for these patients in achieving the targeted IOP and preventing them from having more than one surgery in the future.

A new randomized, controlled study that compares the effects of primary tube versus primary trabeculectomy in refractory glaucoma is currently recruiting participants through the Bascom Palmer Eye Institute (ClinicalTrials.gov Identifier: NCT00666237). Due to the bias of our retrospective study and a lack of evidence regarding the primary choice for refractory glaucoma patients in the Chinese population, multi-center randomized controlled trials are needed in the future.

## Supporting Information

S1 Data Supplemental data. Baseline characteristics and clinical course for FP7/8 AGV implant.
(XLSX)
